# Surface modification of activated carbon cloth with calcium silicate and hydroxyapatite: bioactive composite material

**DOI:** 10.1016/j.heliyon.2022.e11586

**Published:** 2022-11-12

**Authors:** Yohana Y. García-Guel, Elia M. Múzquiz-Ramos, Jorge C. Ríos-Hurtado, Anastasio Moreno-Santos, Sergio E. Flores-Villaseñor, Griselda B. Escalante-Ibarra

**Affiliations:** a Facultad de Ciencias Químicas, Universidad Autónoma de Coahuila, Blvd. V. Carranza y J. Cárdenas Valdés s/n, 25280, Saltillo, Coahuila, Mexico; b Facultad de Metalurgia, Universidad Autónoma de Coahuila, 25710, Monclova, Coahuila, Mexico

**Keywords:** Activated carbon cloth, Oxidation, Hydroxyapatite, Bioactive composite, Bone regeneration

## Abstract

In this study new compounds consisting of activated carbon cloths (ACC) modified with calcium silicate (CaSiO_3_) were prepared for hydroxyapatite (HAP) generation. ACC samples were oxidized with 8 M HNO_3_ at different times (15 min and 2 h), to increase oxygenated functional groups. The CaSiO_3_ fine powders were prepared by chemical coprecipitation using Ca(NO_3_)_2_∙4H_2_O and Si(OC_2_H_5_)_4_, and 5 M NaOH was used as precipitant. The resulting powders were mixed with ethanol by ultrasound stirring and the previously oxidized activated carbon fibers were placed leaving under stirring for 30 min to allow particle dispersion. Once the formed compounds were dried, the samples were immersed in a simulated body fluid (SBF) solution for 21 days in conical tubes at 36.5 °C to allow the HAP formation on the ACC/CaSiO_3_ composite surface. The results indicated that the increase in oxidation time improves HAP formation on the surface from ACC/CaSiO_3_ compounds and this bioactive composite may be a potential material for bone regeneration.

## 1 Introduction

In recent years, medical treatments cost for traumatisms, infections and tumors related to bone tissue, has increased since there is a growing need for repair or replacement of damaged tissues for healthy ones [1, 2, 3]. Bone is composed by a calcified osseous matrix, cells, and bioactive compounds. The matrix mass contents around 69 % of mineral materials, 22 % of organic compounds and 9 % of water [4]. Hydroxyapatite (HAP), Ca_10_(PO_4_)_6_(OH)_2_, is the principal mineral inorganic phase [5, 6] with a Ca/P ratio less than 1.67 [7,8]. It is considered the most adequate bioceramic material for biomedical applications [9, 10] due to its osteogenic potential that helps in bone growth and tissue adhesion [11].

More than 500,000 procedures of bone graft are performed to treat fractures and other orthopedics related traumatisms resulting in diverse degenerative and traumatic conditions [12]. However, actual clinical treatments for bone injuries such as autografts, allografts and xenografts are not used because of the potential risks of disease transmission, infection and host rejection [1, 12, 13, 14]. Also, several materials commercialized are not bioactive, osteoinductor and have a high risk of producing bacterial infections [15, 16, 17]. For this reason, new materials and solutions are being developed to satisfy medical needs, where it can stand out a trend using polymeric, ceramic and composites materials instead of metals [1, 15, 18, 19].

Fibers and textiles have been used for medical applications especially in regenerative medicine. Human body is full of fibers which is suitable for cells adaptation and management. The principal advantage of textiles is its mechanical properties, flexibility and elasticity, as well as potential designs and structures [20]. Activated carbon fiber (ACF) is a new activated carbon form. The growing attention of carbon in all its forms is due to biocompatibility with live tissues [21]. Garcia-Ruiz et al. developed a novel biomimetic scaffolds system. It is based on structure manufacturing designed by computer, in which ACF is knitted or incorporated in order to be functionalized with mesenchymal stem cells (MSC) to stimulate chondrogenesis [22]. Boehm et al. improved calcium phosphate cement (CPC) mechanical properties of injectable and load-bearing areas bones substitutes by incorporating ACF chemically modified with different oxidizing agents [23].

There are two ACF forms: cloth and felt [24, 25]. Activated carbon fiber felt has a lower density than the cloth, making it suitable for low weight applications, such as water and chemical treatment, and for protection masks. For activated carbon cloth (ACC) has high porosity [26], high surface area (1000–2100 m^2^ g^−1^), small fiber diameter, faster adsorption kinetics, average micropore size distribution, and a higher adsorption capacity for low concentration of adsorbate than other adsorbent materials [27]. These characteristics make suitable ACC to be used for several applications like in medicine, because of the biocompatibility of the material [28, 29]. Lopez Peñalver et al. demonstrated that ACC is a good support for MSC, reducing their growth time and improving their proliferation and differentiation [30]. Ruiz de Almodóvar et al. patented a new biomaterial that allows bone tissue and artificial bone regeneration by stem cells from umbilical cord previously differentiated on a ACC matrix [31].

In the present work, a methodology to obtain an ACC/CaSiO_3_/HAP composite is presented. ACC samples were oxidized with HNO_3_ varying time. Also, CaSiO_3_ was added to improve the HAP formation on the composite surface using simulated body fluid (SBF).

## 2 Methods

### 2.1 Activated carbon cloth (ACC) oxidation

Activated carbon cloth FM10 (Flexzorb™ ACC) was provided by Calgon Carbon Corporation. ACC modification was performed using HNO_3_ as an oxidizing agent, as reported by Rangel-Mendez and Streat [32]. In a ball flask, 50 mL of HNO_3_ in an 8 M concentration was heated at 85 °C. Once the temperature was constant, a 3 cm^2^ ACC piece was added to the flask, varying the oxidation time in 15 min (CC15m) and 2 h (CC2h). The time was assigned with a low and high contact time with the oxidizing agent, by preliminary tests. A piece without oxidation process was used as control (CCSOx). After the oxidation time was concluded, the flask was submerged in cold water in order to stop the reaction. Finally, samples were washed with sufficient deionized water and dried at room temperature for 48 h.

### 2.2 Calcium silicate (CaSiO_3_) synthesis and dispersion on ACC

Calcium silicate powders were prepared by coprecipitation method using CaNO_3_ ∙ 4H_2_O (Fermont) and TEOS (Sigma-Aldrich) as raw materials. The compounds were dissolved separately in ethanol. When dissolved, both solutions were mixed and the concentration was adjusted to 0.2 mol L^−1^, according to the method reported by Hazar [33]. The solution was mixed by magnetic stirring for 30 min. Precipitation was performed adding a NaOH 5 M solution. The precipitate obtained was filtered (Whatmann® filter paper No. 42) and dried at 100 °C for 12 h and finally the product was calcined at 800 °C during 2 h.

CaSiO_3_ powders were dispersed on the ACC, using an ultrasonic bath. CCSOx, CC15m and CC2h were submerged separately in a Petri dish with 20 mL of ethanol, and 0.1 g of CaSiO_3_ powders were dropped in the solution and mixed in an ultrasonic bath for 30 min at room temperature, for a dispersion on the ACC surface. All samples were shaken to remove excess of calcium silicate powder, The dispersion carried out was stable at the conditions of the experiments since no CaSiO_3_ particles were observed in the remaining SBF solution. Finally, the resulting products were dried at room temperature for 48 h.

### 2.3 Hydroxyapatite (HAP) generation with simulated body fluid (SBF)

HAP formation on the ACC/CaSiO_3_ samples (CCSOx, CC15m, CC2h) surface was performed using SBF according to the method proposed by Kokubo and Takadama [34]. The solution was prepared according to the body ions concentration (Table 1) [33,35]. Once the solution was prepared, 20 mL were added to a conical tubes bottle and the ACC/CaSiO_3_ samples were added separately. Conical tubes were sealed and put in an incubator (LabTech LSI-3016A) at 36.5 °C for 21 days. Finally, the samples were washed with deionized water and dried at room temperature.

**Table 1:** Ion concentrations of simulated body fluid and human blood plasma.

Ion	Ion concentration (mM)	
	Blood plasma	SBF
Na^+^	142	142
K^+^	5	5
Ca^2+^	2.5	2.5
Mg^2+^	1.5	1.5
HCO_3_^-^	27	4.2
Cl^-^	103	148.5
HPO_4_^2-^	1	1
SO_4_^2-^	0.5	0.5

### 2.4 Materials characterization

In order to verify HAP growth, the products obtained were characterized by Field Emission Scanning Electron Microscopy with EDS (JEOL JSM-7200F) without previous preparation since the samples were conductive, Infrared spectroscopy with ATR module and KBr preparation (PerkinElmer Nicolet Nexus 47), X-ray Photoelectron Spectroscopy (Thermofisher Scientific K-Alpha), Thermogravimetric Analysis with and oxidizing air atmosphere (TA Instruments TGA 550) and N_2_ physisorption at 77 K (BEL BELSORP-miniX). Boehm titrations were carried out according to Schönher et al [36]. XRD measurements were made on Bruker D8 Advance equipment (CuKa: 1.54 Å, 40 mA and 40 kV).

## 3 Results and discussion

### 3.1 ACC/CaSiO_3_/HAP thermal, texture, and morphological properties

#### 3.1.1 Thermal properties

**Figure 1:**
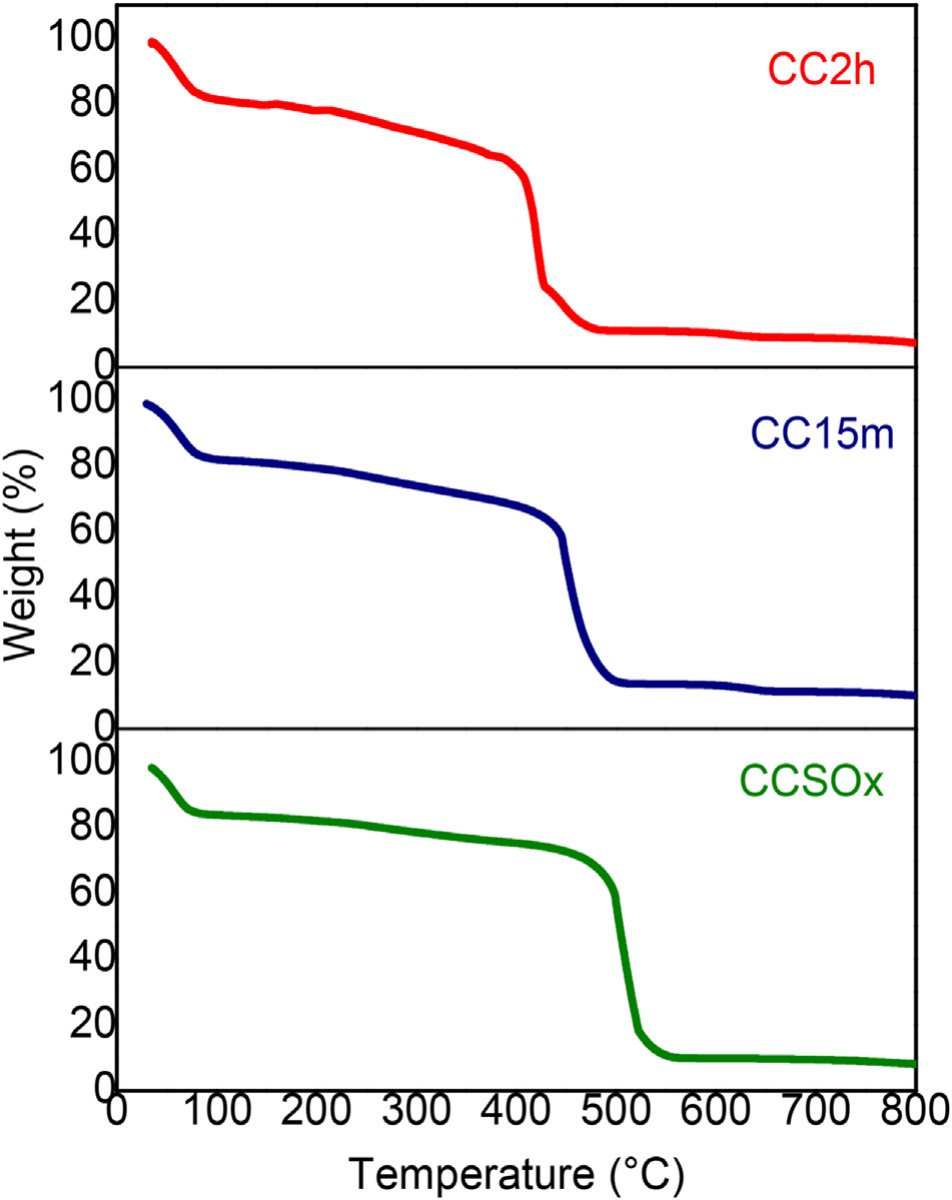
TGA of modified ACC with CaSiO_3_ after 21 days of immersion in SBF.

Thermogravimetric analysis was carried out to determine ACC decomposition temperature and oxidation degree. Figure 1 shows the TGA curves at 10 °C min^−1^ heating rate for CCSOx, CC15m and CC2h samples, which showed a similar behavior. The first drop observed was at 80 °C for all the samples, attributed to moisture loss from ACC surface.

Moreover, the difference in degradation temperatures in the next thermal decomposition observed was of 90 % loss due to carbon oxidation and oxygen loss. The degradation temperature for CCSOx was 510 °C, 461 °C for CC15m and 422 °C for CC2h. The difference in the samples is the time in contact with the oxidizing agent and can be inferred that when the oxidation degree increases the degradation temperature decreases due to the presence of more oxygenated groups, which has been reported that improves the combustion process [37]. Furthermore, the remaining 10 % in the samples, increasing with oxidation time 7.83% for CCSOx, 8.91% for CC15m and for CC2h 9.19%, that was attributed to the presence of inorganic materials, such as HAP, CaSiO_3_ and a minimum amount of ashes (<5 %) [38]. The results demonstrated the presence of an inorganic materials with a higher amount increasing the oxidation time.

#### 3.1.2 Morphology properties

**Figure 2:**
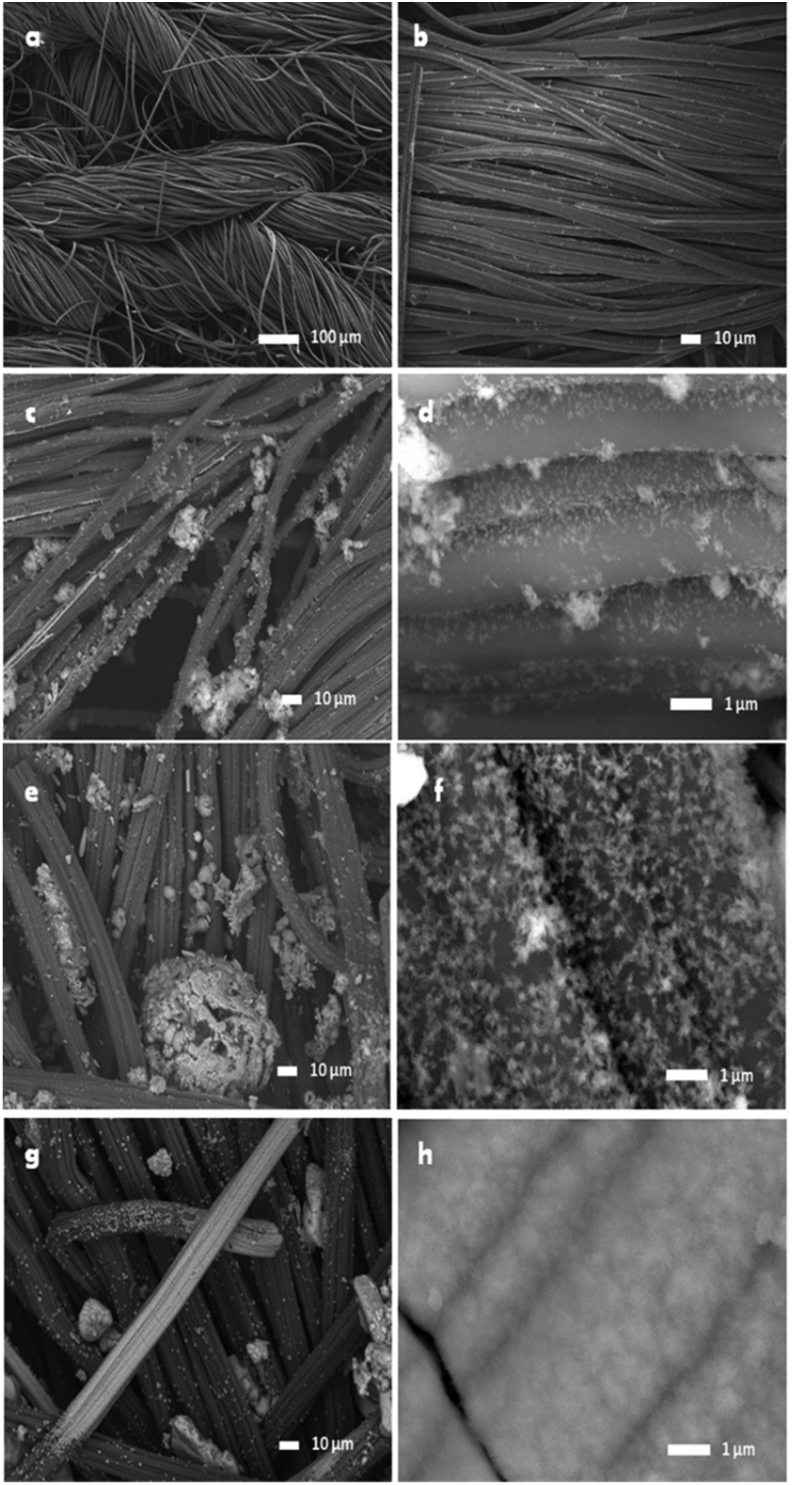
SEM images: commercial ACC (a,b); CCSOx after 21 days in SBF (c, d); CC15m after 21 days in SBF (e, f); CC2h after 21 days in SBF (g,h).

Surface morphology of the samples was evaluated by scanning electron microscopy technique. Figure 2 shows the micrographs of the analyzed samples. Commercial ACC is shown in Figures 2a and 2b, where an arrangement in the fabric fibers is clearly observed. In addition, each fiber is formed by several filaments of the original precursor material (rayon). ACCs modified with CaSiO_3_ and immersed in SBF for 21 days showed different surface structures; in CCSOx (Figures 2c and 2d) and in CC15m (Figures 2e and 2f) the formation of small distributed particles can be observed, which is attributed to the formation of HAP after immersion in SBF. Figures 2g and 2h shows the CC2h, in which in contrast to the previous samples, a greater HAP growth can be observed on a large part of the fibers. As described above, it can be inferred that CC2h presents higher bioactivity since the HAP formation on the surface of the material is clearly observed (Figure 2h) indicating that, the increase in oxidation time improves the HAP formation.

#### 3.1.3 Texture properties

The texture measurements were made with the N_2_ physisorption 77 K technique, in BEL BELSORP-mini-X equipment. CC2h texture properties are summarized in Table 2. The surface area of the conventional ACC and CC2h are 1300 and 1012 m^2^ g^−1^ and the pore volumes are 0.439 and 0.491 cm^3^ g^−1^, respectively. The results show that the oxidation caused a decrease in the surface area. This behavior can be attributed to the destruction or combustion of the pore walls, and to the narrow pore blockage caused by the products formed during the oxidation or by the introduction of oxygenated groups on the surface [39]. A slight increase in the pore volume of the CC2h sample is observed, which may be due to the presence of CaSiO_3_ and HAP which are porous particles [40]. The average pore diameter for conventional ACC was 0.22 nm and for CC2h was 1.94 nm, which shows that there was a small increase because acid oxidation slightly enlarges the pores [32]. The porous structure consists mainly of micropores because the average diameter is less than 2 nm [41, 42]. The presence of the SBF ions that begin to form the hydroxyapatite particles seems to clog the pores, so that a decrease in their surface area and pore volume values is observed in the samples CCSOx and CC15m.

**Table 2:** Texture properties of the samples.

Sample	A _sup_	V _pore_	D _pore_
	(m^2^ g^−1^)	(cm^3^ g^−1^)	(nm)
Commercial ACC [43]	1300	0.439	0.22
CCSOx	809	0.401	2.43
CC15m	741	0.398	2.65
CC2h	1012	0.491	1.94

**Figure 3:**
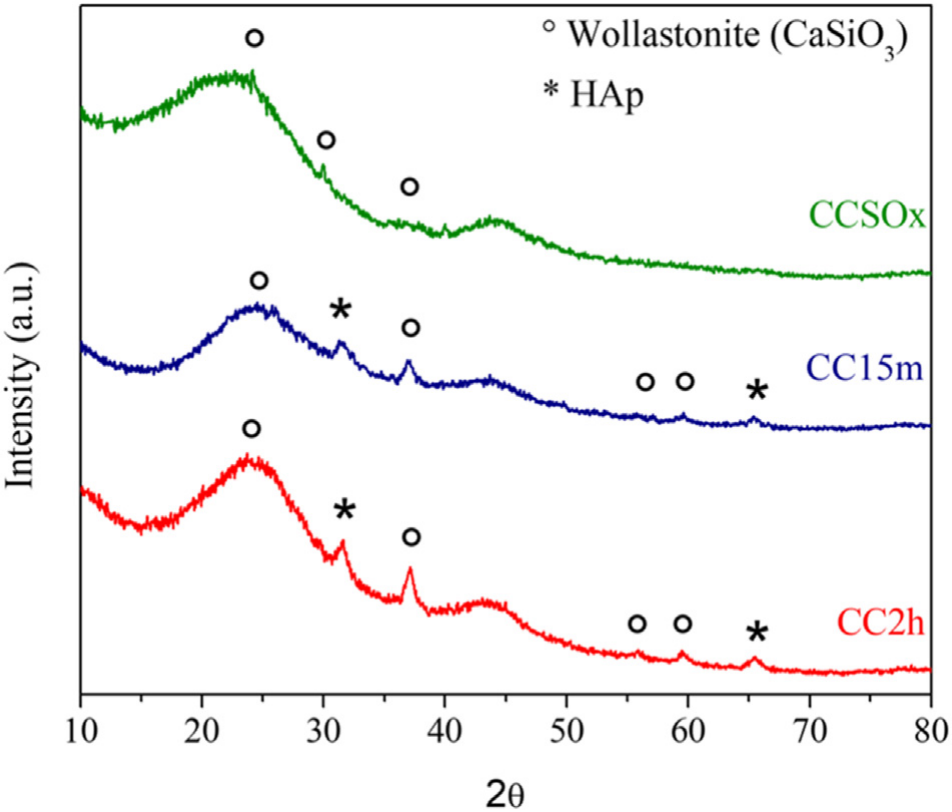
XRD patterns for the incubated samples with SBF and CaSiO_3_.

Figure 3 shows the XRD patterns for the obtained samples. The results demonstrated the presence of HAP on ACC surface observed in the signals around 32°, and 66° for CC2h and CC15m samples corresponding to JCPDS 96-901-1095 and also signals at 24°, 28°, 36° and 56° corresponding to CaSiO_3_ were observed in all the samples (JCPDS 96-901-1095). For CCSOx any signal was observed for HAp. The typical amorphous graphite surface was observed on signals between 20°–30° and 40°–50°. The intensity of the signals depends on the amount of the ceramic materials, and only 5% of the composite were of these.

#### 3.1.4 ACC/CaSiO_3_/HAP chemical properties

**Figure 4:**
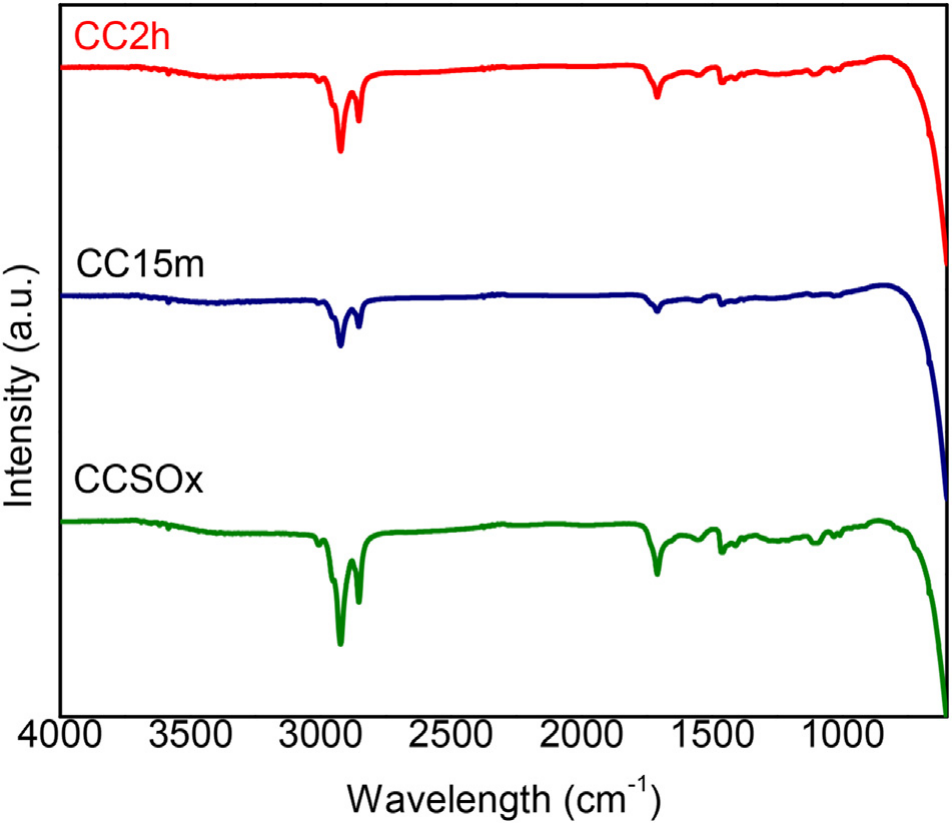
FTIR spectra for modified ACC with CaSiO_3_ after 21 days in SBF.

To determine the bonds formed after ACC modification, infrared spectroscopy tests were performed. Figure 4 shows the infrared spectra for CCSOx, CC15m and for CC2h. All the samples analyzed revealed very similar spectra, where the most significant double signal is located between 2800 and 3000 cm^−1^ and corresponds to the C–H stretch. The band at 1715 cm^−1^ is attributable to the C=O stretch of the carbonyl group, and the band at 1456 cm^−1^ corresponds to methyl group (-CH_2_) bending vibrations. These bands are characteristic for carbon materials, and although there is no clear difference between the studied samples, it has been proven in previous studies that by contacting an activated carbon with an oxidizing agent such as nitric acid, the amount of oxygenated groups concentration increases [44]. Also, small signals in 1116 and 1085 cm^−1^ corresponds to the phosphate ion present on the HAp structure [45]. The typical signals of wollastonite were not observed since all the particles were coated with hydroxyapatite, and is important to note, that since the activated carbon cloth is the 95% content of the material, the functional groups of hydroxyapatite are less intense than the ones of ACC.

To demonstrate the successful activation of the surface, titrations were carried out and the results are shown in Table 3, in comparation with the oxidized materials, ACC (without oxidation) ACC15m (15 min oxidation) and ACC2h (2 h oxidation). The commercial ACC showed the presence of carboxyl (0.075 meq·g^−1^) and lactones (0.124 meq·g^−1^) groups, due to the carbonization and activation process. When the commercial ACC was added to the oxidizing agent (HNO_3_) varying time, the oxygenated functional groups were increased on the surface of the samples [44]. For the incubated samples with CaSiO_3_ and SBF solution (CCSOx, CC15m, CC2h), the quantity of oxygenated groups decreased. The results demonstrate that oxygenated groups are a determining factor in the formation and growth of hydroxyapatite on the surface of the activated carbon cloth. The carboxyl groups showed the highest decrease and by this it can be inferred that with a higher presence of these groups the growth of hydroxyapatite will be enhanced.

**Table 3:** Oxygenated functional groups quantification (meq·g^−1^) on the samples surface obtained by titrations.

Sample	Carboxyl	Lactones	Phenols
ACC	0.075	0.124	N/D
CCSOx	0.071	0.119	N/D
ACC15m	0.242	0.299	0.043
CC15m	0.225	0.288	0.041
ACC2h	0.398	0.376	0.108
CC2h	0.371	0.365	0.101

∗ND: Not detectable.

**Figure 5:**
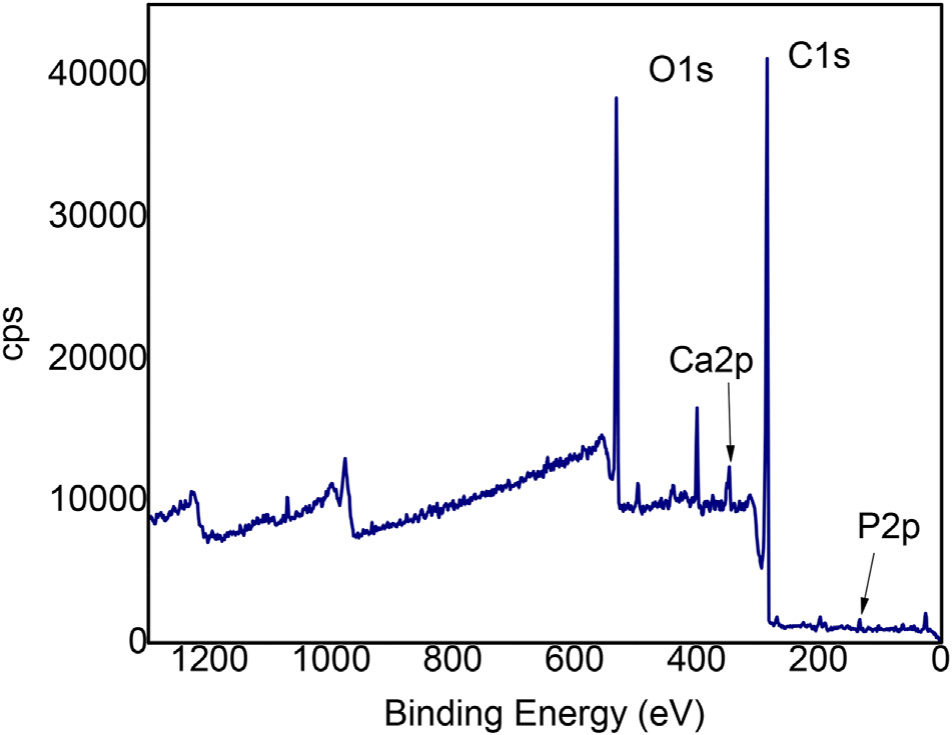
XPS analysis of CC2h after 21 days of immersion in SBF.

Moreover, an XPS analysis was performed to understand the chemical composition of CC2h, which shows the higher amount of HAP formation. Figure 5 shows XPS survey indicating the presence of signals corresponding to C1s, O1s, due to the oxidized ACC, and the Ca2p and P2p signals, which is attributed to the HAP presence on the analyzed surface.

**Figure 6:**
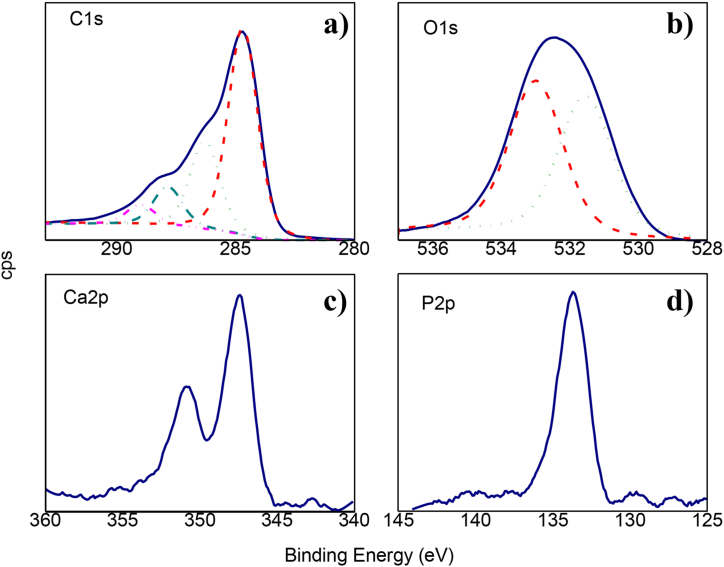
XPS spectra of CC2h: (a) C1s; (b) O1s; (c) Ca2p; (d) P2p.

In addition, the elements spectra are shown in Figure 6, and the binding energy results in Table 4. The C1s spectrum corresponding to carbon (Figure 6a) has four components with chemical shifts attributed to the graphitic layer (284.6 eV) and the oxygenated groups: hydroxyl or phenol (286.3 eV), carbonyl (287.8 eV) and carboxyl groups (288.9 eV) [46]. The O1s region corresponding to oxygen (Figure 6b) fits in two components, located at 531.5 eV, due to double bonds -C=O in carboxylic acids and ketones, while the peak at 533.0 eV is assigned to simple -C–O in phenols, alcohols, and carboxylic acids, corresponding to those seen in the carbon region [47]. For calcium (Figure 6c), the typical bands of Ca2p_3/2_ and Ca2p_1/2_ located at 347.5 and 350.8, respectively, are shown. The presence of Ca2p_3/2_ suggests that CC2h contains HAP particles formed after immersion in SBF for 21 days. This is consistent with the signal for phosphorus (Figure 6d), which is found in 133.6 eV and corresponds to P2p_3/2_ from hydroxyapatite [48].

**Table 4:** Elements determined by XPS spectra for CC2h.

Element	Binding Energy (eV)	Attribution	Reference
**C**	284.6	C=C	[47]
	286.3	C–O	
	287.8	C=O	
	288.9	O–C=O	
**O**	531.5	C=O	[28]
	533	C–O	
**Ca**	347.5	Ca2p_3/2_	[48]
	350.8	Ca2p_1/2_	
**P**	133.6	2p_3/2_	[18]

EDS analysis was performed to determine the elemental composition and the Ca/P ratio in CC2h sample. The results in Table 5 show that the ACC is mainly constituted of carbon and oxygen and contains a small number of other elements corresponding to the ions used in the SBF preparation. In addition, CC2h contains a 17.85 % calcium mass and 8.68 % phosphorous mass. These results demonstrate HAP formation on the ACC surface because Ca/P ratio of 1.59, in general, the theoretical value of the Ca/P ratio for HAP is 1.67 [49].

**Table 5:** Elemental analysis of ACC modified with CaSiO_3_ after 21 days in SBF.

Sample	Elements (% weight)									
	C	O	Na	P	Cl	Ca	Mg	Al	Si	Zn
CCSOx	40.46	38.79	ND	2.09	1.21	9.21	0.21	1.93	3.25	2.86
CC15m	19.46	53.51	0.22	0.62	0.20	25.65	0.33	ND	ND	ND
CC2h	14.98	46.91	2.04	8.68	2.97	17.86	ND	ND	6.55	ND

∗ND: Not Detectable.

## 4 Conclusions

As observed in SEM images, CC2h (oxidized for 2 h) has a higher HAP content when immersed in SBF for 21 days. The EDS analysis showed a mass of 17.85 % for Ca and 8.68 % for P in CC2h, which indicates a 1.59 ratio for Ca/P. These results agree with the EDS analysis, showing Ca2p and P2p signals, corresponding to the HAP presence on the analyzed surface, results observed also in XRD patterns. In addition, TCA2h lost more than 90 % of their weight at a temperature of 422 °C and around 5 % weight corresponds to HAP and CaSiO_3_. Oxidation causes a slight decrease in surface area and a large part of the porous structure consists mainly of micropores (<2 nm). The bioactivity tests showed that the compounds (ACC/CaSiO_3_) improve the HAP growth on the ACC surface with a higher oxidation degree. This compound may be a potential material for bone regeneration, however tight adhesion and the mechanical properties of the samples need to be measured.

## Declarations

### Author contribution statement

Yohana Y. García-Guel: Performed the experiments; Analyzed and interpreted the data; Wrote the paper.

Elia M. Múzquiz-Ramos: Conceived and designed the experiments; Analyzed and interpreted the data.

Jorge C. Ríos-Hurtado: Conceived and designed the experiments; Analyzed and interpreted the data; Wrote the paper.

Anastasio Moreno-Santos: Performed the experiments.

Sergio E. Flores-Villaseñor: Analyzed and interpreted the data.

Griselda B. Escalante-Ibarra: Contributed reagents, materials, analysis tools or data.

### Funding statement

This work was supported by Consejo Nacional de Ciencia y Tecnología (CONACyT), México, Grant 440931 and “Proyecto Semilla” CGEPI-UADEC C01-2019-06.

### Data availability statement

Data included in article/supplementary material/referenced in article.

### Declaration of interests statement

The authors declare no conflict of interest.

### Additional information

No additional information is available for this paper.
